# Recovery from desensitization in GluA2 AMPA receptors is affected by a single mutation in the N-terminal domain interface

**DOI:** 10.1016/j.jbc.2024.105717

**Published:** 2024-02-02

**Authors:** Andreas Haahr Larsen, Amanda M. Perozzo, Philip C. Biggin, Derek Bowie, Jette Sandholm Kastrup

**Affiliations:** 1 Department of Neuroscience, University of Copenhagen, Copenhagen, Denmark; 2 Department of Pharmacology and Therapeutics, McGill University, Montreal, Canada; 3 Structural Bioinformatics and Computational Biochemistry, Department of Biochemistry, University of Oxford, Oxford, UK; 4 Department of Drug Design and Pharmacology, University of Copenhagen, Copenhagen, Denmark

**Keywords:** GluA2, GluA1, AMPAR, electrophysiology, patch clamp, small-angle neutron scattering, SANS, molecular dynamics, MD, metadynamics, potential of mean force

## Abstract

AMPA-type ionotropic glutamate receptors (AMPARs) are central to various neurological processes, including memory and learning. They assemble as homo- or heterotetramers of GluA1, GluA2, GluA3, and GluA4 subunits, each consisting of an N-terminal domain (NTD), a ligand-binding domain, a transmembrane domain, and a C-terminal domain. While AMPAR gating is primarily controlled by reconfiguration in the ligand-binding domain layer, our study focuses on the NTDs, which also influence gating, yet the underlying mechanism remains enigmatic. In this investigation, we employ molecular dynamics simulations to evaluate the NTD interface strength in GluA1, GluA2, and NTD mutants GluA2-H229N and GluA1-N222H. Our findings reveal that GluA1 has a significantly weaker NTD interface than GluA2. The NTD interface of GluA2 can be weakened by a single point mutation in the NTD dimer-of-dimer interface, namely H229N, which renders GluA2 more GluA1-like. Electrophysiology recordings demonstrate that this mutation also leads to slower recovery from desensitization. Moreover, we observe that lowering the pH induces more splayed NTD states and enhances desensitization in GluA2. We hypothesized that H229 was responsible for this pH sensitivity; however, GluA2-H229N was also affected by pH, meaning that H229 is not solely responsible and that protons exert their effect across multiple domains of the AMPAR. In summary, our work unveils an allosteric connection between the NTD interface strength and AMPAR desensitization.

AMPA-type ionotropic glutamate receptors (AMPARs) are critical signaling components of excitatory synapses, facilitating sodium influx and membrane depolarization upon binding of the neurotransmitter, L-glutamate. AMPARs are also pivotal in various neurological processes, such as memory and learning (1, 2), and are implicated in neuropathogenic diseases (3) as well as stroke recovery (4).

AMPARs exist as stable homo- or heterotetramers comprising the subunits GluA1, GluA2, GluA3, and GluA4. Each subunit consists of an N-terminal domain (NTD), a ligand-binding domain (LBD), a transmembrane domain (TMD), and a C-terminal domain (CTD) (Fig. 1*A*). The gating mechanism of AMPARs is governed by a reconfiguration of the LBD layer (5, 6) upon agonist binding. The NTD of AMPARs contributes to tetramer assembly (7), synaptic anchoring (8), regulation of dendritic spine density (9, 10), and intrinsic mobility. During the gating cycle, the NTD displays dynamic behavior, with NTD splaying occurring in the desensitized state (11, 12, 13) (Fig. 1*B*).

**Figure 1:**
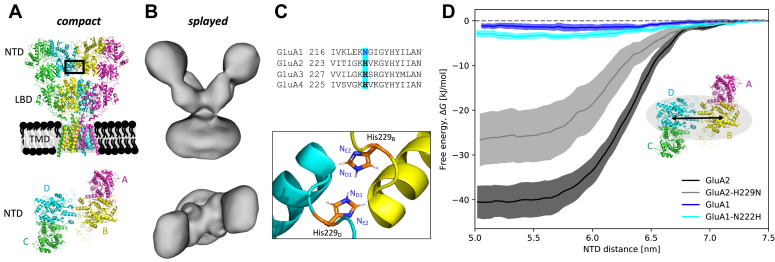
**NTD interface in GluA1, GluA2, GluA2-H229N and GluA1-N222H**. *A*, GluA2 in the desensitized compact state (PDB: 5VHZ (18), auxiliary units omitted). NTD: N-terminal domain, LBD: ligand-binding domain, TMD: transmembrane domain. GluA2 also contains an intrinsically disordered C-terminal domain, which has been omitted in the EM structure. *B*, GluA2 in the desensitized NTD-splayed state (EMDB-2688 (11)). *C*, sequence alignment of GluA1-4 NTD residues involved in formation of the dimer-of-dimer interface, highlighting H229 in GluA2 and N222 in GluA1. The H229 pair at the GluA2 NTD interface is shown. *D*, NTD subunits at positions B and D (insert) were isolated *in silico* and pulled apart to estimate the free energy of NTD binding. Free energy curves (mean and standard errors) as a function of NTD distance (*black arrow* in insert) were calculated for GluA1 (*blue*), GluA1-N222 (*light blue*), GluA2 (*black*), and GluA2-H229 (*gray*).

In native AMPARs, the B and D positions (Fig. 1*A*) are predominantly occupied by GluA2, and the NTD dimer-of-dimers assembly is disfavored if GluA1 occupies these positions (14, 15). We wished to investigate whether the stability of the dimer interface has a connection to desensitization. It is known that the NTDs allosterically affect desensitization, as deletion of NTDs in GluA1-4 reduces rates into densitization (16). Moreover, insertion of the NTD from GluA2 into GluA1 leads to faster recovery, and vice versa (15). However, the NTD-deleted GluA1 homotetramers still have a much faster entry into desensitization and slower recovery from desensitization than NTD-deleted GluA2 (16), so the difference in desensitization between GluA1 and GluA2 is primarily attributed to differences in the affinity of the agonist for the LBD. Interestingly, the effect of NTD deletion is more pronounced in GluA1 (ca. 2.3-fold faster recovery from desensitization after NTD deletion) compared to GluA2 (ca. 1.3-fold faster recovery) (16). The splayed NTDs of GluA1 (15) exert more strain on the LBD than the compact NTDs of GluA2, which may explain why NTD deletion in GluA1 has a larger impact on recovery than in GluA2.

In this study, we employed molecular dynamics (MD) simulations to evaluate the strength of the NTD interface in GluA1 and GluA2 and found that GluA1 has a weaker NTD interface, consistent with the splayed NTDs observed in GluA1 homotetramers (15). Moreover, we demonstrate that weakening of the NTD interface in GluA2 leads to slower recovery from desensitization. This weakening was introduced by mutating H229, which has been suggested to play a crucial role in NTD stabilization (14). H229 is conserved in GluA2-4 but is an asparagine in GluA1 (Fig. 1*C*). The GluA2-H229N mutant thus has a more “GluA1-like” NTD interface. Using electrophysiology, we showed that this mutant also displayed slower recovery from desensitization.

Given the pH sensitivity of histidine, we investigated a potential connection between pH and NTD splaying. Our previous studies, using small-angle neutron scattering (SANS), demonstrated that reduced pH induced NTD splaying in GluA2 in the presence of AMPA (12). In the present study, we reanalyze the SANS data using MD simulations and refine an ensemble of compact and NTD-splayed structures, consistent with the SANS data and previous electron microscopy (EM) findings (11, 13). We thus argue that desensitized AMPARs exist in an equilibrium between compact and NTD-splayed states, and that this equilibrium is shifted by changes in pH. Employing electrophysiology, we demonstrated that protons enhance desensitization, in line with previous studies (17). Desensitization was, however, also enhanced in the GluA2-H229N mutant at lower pH. Therefore, the H229 site alone cannot explain the effect of pH.

In summary, we argue that an allosteric connection exists between the strength of the NTD dimer-of-dimers interface and the desensitization of AMPARs. A weaker interface leads to longer recovery times from desensitization, which should be overcome to undergo structural reconfiguration.

## Results

### GluA1 exhibits a weaker NTD dimer-of-dimer interface compared to GluA2

Zhao *et al.* suggested that the NTD interface of AMPARs is disfavoured if GluA1 occupies one or both of the B and D positions (14). To investigate this, we isolated the B and D subunits of the NTDs of GluA1 and GluA2 *in silico* and estimated the free energy of NTD-binding. The binding energy was −40 ± 4 kJ/mol for GluA2 and significantly lower at −3.9 ± 1.1 kJ/mol for GluA1 (Fig. 1*D* and Table 1), indicating a substantial weakening of the NTD interface when positions B and D are occupied by GluA1.

**Table 1:** Desensitization rates (entry and recovery) and free energy of NTD binding. $\tau_{\text{entry}}$ is the entry rate from the open to desensitized state; $\tau_{\text{recov}}$ is the recovery rate from desensitized to resting state

Neutral pH 7.4	$\tau_{entry}$ [ms] (*n*)	$\tau_{recov}$ [ms] (*n*)	ΔG [kJ/mol] (*n*)
Construct			
GluA1	3.1 ± 0.2 (6)	166 ± 8 (4)	−1.6 ± 0.7 (5)
GluA1-N222H	2.9 ± 0.1 (5)	160 ± 10 (5)	−3.6 ± 0.6 (5)
GluA2	8.9 ± 0.4 (15)	17 ± 1 (7)	−40 ± 4 (5)
GluA2-H229N	7.3 ± 1.4 (13)	30 ± 1 (8)	−26 ± 6 (5)

Acidic pH 5.5	$\tau_{entry}$ [ms] (*n*)	$\tau_{recov}$ [ms] (*n*)	Peak ratio (pH 5.5/7.4) (*n*)
Construct			
GluA2	4.5 ± 0.3 (15)	75 ± 11 (6)	12 ± 1% (15)
GluA2-H229N	3.8 ± 0.2 (12)	61 ± 6 (5)	12 ± 1% (12)

For neutral pH 7.4: ΔG is the free energy of binding between the NTD at position B and the NTD at position D. For acidic pH 5.5: Peak ratio of glutamate-induced current peaks. Mean and standard errors of *n* repeats.

### Weakening the NTD interface of GluA2 by a single-point mutation

Zhao *et al.* further suggested that H229 (numbering from UniProt entry P19491: GRIA2_RAT) is important for NTD stabilization (14). H229 is conserved in GluA2-4, whereas GluA1 features an asparagine at this site (N222, UniProt entry P19490: GRIA1_RAT) (Fig. 1*C*). As H229 is an N in GluA1 (Fig. 1*C*), we made the H229N mutation, which weakened the NTD dimer-of-dimer interface *in silico* (Fig. 1*D* and Table 1). In contrast, GluA1-N222H exhibited only a very modest increase in NTD binding energy compared to wildtype GluA1.

### Weakening of the NTD interface affects recovery from desensitization

**Figure 2:**
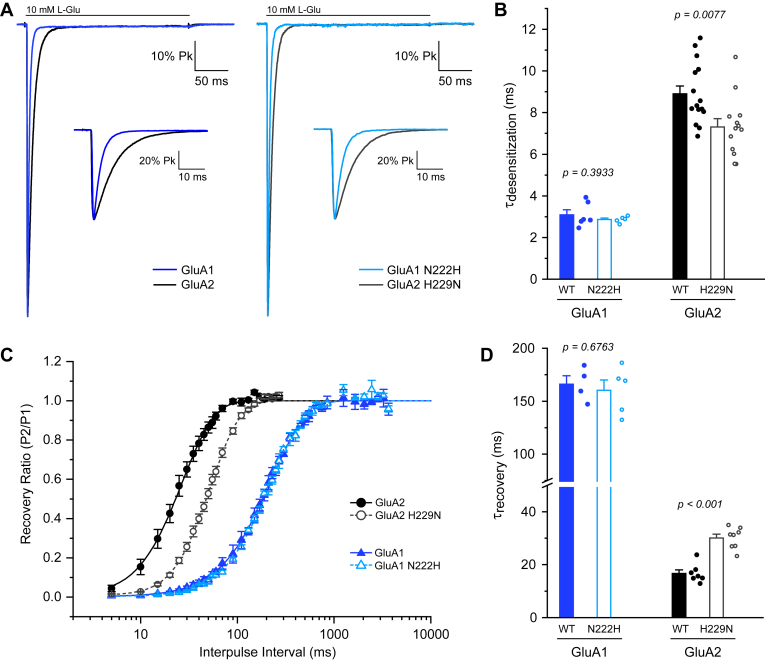
**GluA2-H229N has a slower recovery from desensitization**. *A*, entry into desensitization for GluA2 (*black*), GluA1 (*blue*), GluA2-H229N (*gray*), and GluA1-N222H (*light blue*) receptors in response to long (250 ms) applications of 10 mM L-glutamate. Normalized sample currents from one experiment, where the inset depicts current decay on a shorter time scale. *B*, desensitization time constants for wild types and mutants. *C*, time course of recovery from desensitization for the same constructs. Data is mean ± SEM for 4 to 8 individual recordings. *D*, recovery time constants for all repeats.

Next, we performed electrophysiology recordings to investigate the effect of the mutation on both entry into and exit from desensitization. GluA2-H229N exhibits a slightly faster entry into desensitization (7.3 ± 1.4 ms) than wild-type GluA2 (8.9 ± 0.4 ms) and exhibits a 2-fold slower recovery from desensitization (30 ± 1 ms) compared to wild-type GluA2 (17 ± 1 ms) (Fig. 2 and Table 1). In contrast, the N222H mutation in GluA1 did not affect desensitization, neither on entry nor recovery rates. To summarize, weakening the NTD interface, as achieved with the H229N mutation in GluA2, leads to slower recovery from desensitization.

### Protons induce NTD-splaying in GluA2 in the presence of AMPA

**Figure 3:**
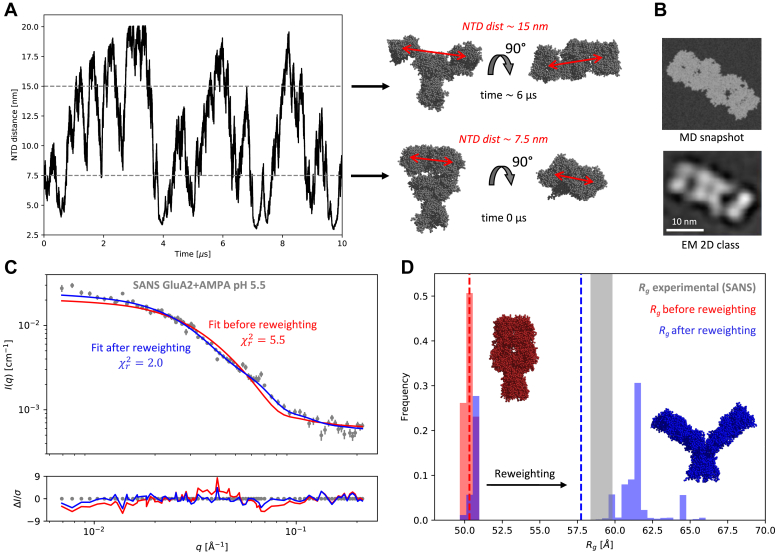
**Protons’ effect on NTD splaying.***A*, NTD distance as a function of time. The NTD distance is here the distance between the center of mass of one NTD dimer to the center of mass of the other NTD dimer of the GluA2 homotetramer. Representative structures are shown. *B*, MD snapshot of GluA2 in the splayed state, compared to a representative EM 2D class of GluA2 with AMPA in the splayed state (13). *C*, Fit to SANS data before and after reweighting of the ensemble. *D*, distribution of radius of gyration, *R*_g_, before and after reweighting, with *dashed lines* marking the mean *R*_g_ values. The *gray area* is the experimental *R*_g_ ± one standard deviation. Representative compact and NTD-splayed structures are displayed.

Using SANS, we have previously reported that GluA2 is affected structurally at lower pH (12). In the presence of AMPA, GluA2 at pH 5.5 exhibited a splayed state, similar to the EM class 3 (EMDB-2688 (11)). In contrast, GluA2 at pH 7.5 and in the presence of AMPA was best described with a compact state (PDB: 5VHZ (18)). However, EM data suggest that GluA2 is likely in equilibrium between states with different degrees of NTD splaying (11, 13), resembling the structural state of homotetrameric GluA1 (15). Therefore, to get a better structural understanding of GluA2, we reanalyzed the SANS data using an ensemble refinement method. Initially, we simulated the GluA2 homotetramer in a lipid bilayer. In the simulations, GluA2 tended to stay in the compact state, as this is the energy minimum of the system. Therefore, we applied metadynamics enhanced sampling to “lift” the simulation out of this energy minimum and explore the conformational space. In metadynamics simulations, an energy penalty, that is, a biasing potential is applied if previously explored states are revisited. The NTD distance was used as a collective variable to map the conformational space (Fig. 3*A* and Video S1). Splayed states similar to those observed in EM (11, 13) were observed in the simulated ensemble (Fig. 3*B*). From the initial metadynamics simulations, weights were assigned to each frame in the simulation, as determined from the inverse of the biasing potential. These weights were used when calculating the effective theoretical scattering from the ensemble. By changing the weights, we obtained an ensemble that was more consistent with the SANS data (Fig. 3*C*). Before reweighting, the most frequent states were compact, but after reweighting against the SANS data, the ensemble was a bimodal distribution of compact and NTD-splayed states (Fig. 3*D*). We note that a continuum of states with radii of gyration (*R*_g_) between 49 Å and 67 Å were present in the ensembles both before and after reweighting (Fig. S1), but many of these states had very low frequency. Our new ensemble-based analysis of the SANS data suggest that, in the presence of AMPA, GluA2 exists in an equilibrium between compact and splayed states. In alignment with EM data of GluA2 in the presence of AMPA (11, 13), protons shift the equilibrium towards the splayed states.

### H229 in the GluA2 NTD is not solely responsible for the pH effect

**Figure 4:**
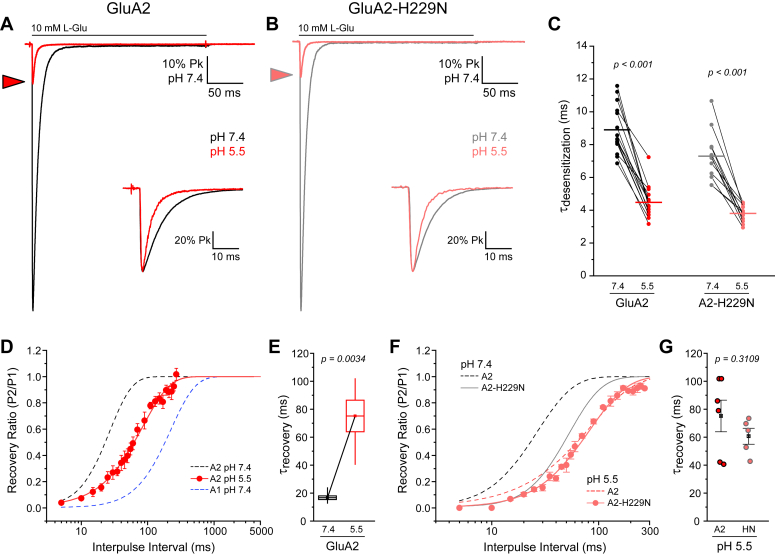
**Protonation effect on desensitization**. *A* and *B*, desensitization of GluA2 and GluA2-H229N at pH 7.4 (*black/grey*) and pH 5.5 (*red/pink*). Example currents from one experiment, with insets depicting normalized current decay on a shorter timescale and the arrowhead indicating the reduction in peak amplitude at pH 5.5. *C*, desensitization time constants for all repeats, with *horizontal lines* depicting the mean. Each patch was exposed to external solution at pH 7.4 and pH 5.5 (*connecting lines*). *D*, time course of recovery from desensitization for GluA2 at pH 5.5 (*red*). Time course of recovery for GluA2 and GluA1 at pH 7.4 shown for comparison (*dashed black* and *blue lines*). *E*, recovery time constants for all experiments, where the box represents the standard error and the whiskers indicate the minimum and maximum values. *F*, time course of recovery for GluA2-H229N at pH 5.5 (*pink dots*). GluA2 and GluA2-H229N at pH 7.4 (*black dashed* and *grey solid lines*) as well as GluA2 at pH 5.5 (*pink dashed line*) shown for comparison. *G*, recovery time constants at pH 5.5 for wild-type GluA2 and GluA2-H229N. *Black square* and error bars indicate mean and standard error, respectively.

Histidine is pH-sensitive and can act as a pH switch (19). Moreover, molecular dynamics demonstrate that if H229 in GluA2 is protonated, the dimer-of-dimer NTD interface is significantly weakened (Fig. S2). However, we estimated H229 to be 80 to 90% buried, resulting in a low effective pKa value. To test experimentally whether H229 is a functional pH switch, we examined the mutant, GluA2-H229N. If the H229 was solely responsible for the pH effect, then this mutant should not be affected by lowering pH. Using electrophysiology, we found that protons enhanced desensitization of GluA2-H229N, resulting in a 2-fold faster entry into desensitization at pH 5.5 (3.8 ± 0.2 ms) compared to pH 7.4 (7.3 ± 1.4 ms). This is similar to the proton sensitivity of wildtype GluA2 (Fig. 4 and Table 1). GluA2-H229N recovery (61 ± 6 ms) is likewise sensitive to protons; however, the mutant is not slowed beyond wild-type GluA2 at pH 5.5 (Fig. 4). For wild-type GluA2, recovery is over 4-fold slower at pH 5.5 (75 ± 11 ms) compared to pH 7.4 (17 ± 1 ms), while for GluA2-H229N recovery is only about 2-fold slower (Table 1). These experiments show that protons do not exclusively rely on H229 to influence desensitization in GluA2, although this site may be involved in proton-mediated slow recovery.

## Discussion

We have investigated the role of the NTD interface strength in AMPAR subunits GluA1 and GluA2 and its connection to desensitization. Before proceeding with our findings, it is important to address the accuracy of our *in silico* methods.

### Accuracy of the MD simulations

The coarse-grained MD simulations have limited accuracy in the description of electrostatics, and are also limited by the application of elastic networks (20). The initial weights on the frames from the metadynamics coarse-grained MD simulation are affected by these limitations. The initial ensemble is therefore not expected to be completely accurate, which is also reflected in an imperfect fit to experimental data before reweighting (Fig. 3*C*). However, the ensemble is “sufficiently good” for reweighting purposes (21, 22), meaning that the conformational space has been sampled to a degree that makes it possible to obtain consistency with experimental data by adjusting the weights on the frames in the simulated trajectory.

To estimate the free energy of splaying, we used the potential of mean force calculations. These were calculated from atomistic simulations on a reduced system, only consisting of two NTD subunits. It is unfeasible to perform umbrella sampling for the whole system with atomistic MD. Therefore, we only included the interfacial NTDs, as these simulations were sufficient to study relative changes.

### NTD splaying allosterically slows recovery from desensitization

Our investigation suggests a link between NTD splaying and recovery from desensitization. We propose that a weakened NTD interface leads to NTD splaying and slower recovery from desensitization. This proposition is supported by the correlation between recovery times and relative NTD interface binding strength (Table 1). Notably, the introduction of the H229N single point mutation in GluA2 weakens the NTD interface, based on our simulations, and substantially slows recovery from desensitization, based on our electrophysiology. The opposite mutation, N222H in GluA1, does not speed up recovery, likely because GluA1 is already splayed (15), and a single mutation is not sufficient to reverse this.

It is worth noting that NTDs are known to impact sodium influx in various ionotropic glutamate receptors. In NMDA receptors, the NTDs act as allosteric sites for modulators (7), and the NTD interface regulates receptor sensitivity and deactivation (23). In Delta receptors, the NTDs require constraints by other proteins for ion conduction (24). The deletion of the NTDs in AMPARs reduces desensitization by slower entry and faster recovery (16). We suggest that in AMPARs, the NTDs can allosterically affect the LBDs and thereby recovery from desensitization. NTD-deleted GluA1 still has a much faster entry and slower recovery than NTD-deleted GluA2 (16), so the difference in desensitization between GluA1 and GluA2 stems mainly from differences at the level of the LBD. However, the same study shows that deletion of the NTDs affects recovery from desensitization significantly, and the effect of NTD deletion is more pronounced in GluA1 (ca. 2.3-fold faster recovery from desensitization) compared to GluA2 (ca. 1.3-fold faster recovery). Moreover, introducing the NTD from GluA2 into GluA1 leads to faster recovery, and vice versa (15). Although the main difference between GluA1 and GluA2 desensitization lies in the LBD, allosteric effects of the NTD also affect recovery from desensitization, with a weaker interface (“GluA1-like”) leading to more strain on the LBD and thereby to slower recovery from desensitization. GluA1 is not found in native GluA2-containing heteromeric AMPARs at the B and D positions (14). This may be explained by the slow recovery rate and weak NTD interface.

### Protons enhance desensitization and lead to more splayed states

We also investigated the role of protons, which shift the conformational equilibrium of GluA2 towards more splayed states. We investigated whether H229 acted as a pH switch, *i.e.*, if protonation of H229 could explain the observed NTD splaying. To test this, we generated the mutant GluA2-H229N. If this mutant was pH-sensitive, the pH effect could not solely be ascribed to this site. We found that GluA2-H229N also reacted to protons, despite lacking the histidine. Furthermore, we estimated H229 to be 80 to 90% buried, resulting in a low effective pKa value. These observations indicate that protons exert their effects across multiple domains, not solely at H229. Intriguingly, the mutant GluA2-H229N was less sensitive to pH-induced slowing of recovery compared to wildtype GluA2, indicating that the H229 site is implicated in pH-induced slowing of recovery. The exact mechanism of pH changes will require substantial future effort and the observations confirm that subtle nuances of control exist for these highly complex receptors.

## Conclusion

AMPARs are vital synaptic ligand-gated ion channels, and their function and structure are, to a large extent, well-described. Nevertheless, while it has been established that the NTD modulates desensitization (16), the precise mechanism remains unclear. Our investigations provide insight into this phenomenon. We argue that NTD splaying stabilizes the desensitized state, *i.e.*, leads to slower recovery from desensitization, by applying strain on the LBD. As postulated by Zhao *et al*. (14), we have shown with electrophysiology that the GluA2-H229N mutant in the NTD dimer-of-dimer interface slows recovery rates from desensitization, and with MD simulations we show that the same mutation weakens the NTD interface.

## Experimental procedures

### Preparing the coarse-grained MD simulations

The crystal structure of full-length homomeric GluA2 in the desensitized state (PDB: 5VHZ (18)) was used as a starting point for the simulation. The structure was missing residues 545 to 572 from all chains. C-terminal residues 818 to 1066 were also missing from chains A, B, and D, and C-terminal residues 821 to 1066 were missing from chain C. The missing residues, up to residue number 832, were added as loops using Modeller (25). The C-terminal residues 833 to 1066 were omitted from the simulations. The C-terminus was also truncated after residue 832 in the construct used for the SANS experiment (GluA2_cryst_ (26), PDB: 3KG2). The protein was oriented according to the Orientations of Proteins in Membranes (OPM) database (27). The protein structure was coarse-grained using the Martinize script (28) to achieve a coarse-grained structure with Martini 3 beads (Martini version 3.0.b.3.2) (29). The protein was placed in a POPC bilayer, in a 34 × 34 × 30 nm^3^ box with periodic boundary conditions, using the insane script (30) (Fig. S3). The system was neutralized with sodium ions and solvated in Martini 3 water (29). All simulations were run in GROMACS 2018.6 (31) with PLUMED (32) patched. The system was first energy-minimized, then equilibrated for 5 ns using 20-fs time steps, Verlet cut-off scheme, van der Waals cut-off of 1.1 nm, and reaction-field electrostatics with a relative dielectric constant of 15. The temperature was kept at 323 K by a v-rescale thermostat for protein, lipids, and solvents, separately, with a time constant of 1 ps. The temperature of 323 K is standard procedure for the simulations with the Martini force field (33). The pressure was kept at 1 bar using a semi-isotropic Berendsen barostat with a time constant of 6 ps and compressibility of 3⋅10^−4^ bar^−1^.

### Coarse-grained MD metadynamics simulations

To force the protein out of the energy minimum (the compact state), we used metadynamics (34, 35), which is an enhanced sampling method that applies an energy penalty if the simulation revisits states. To differentiate between states, we defined a reaction coordinate, being the distance between the center of mass of the NTDs of chain A and B and the center of mass of the NTDs of chain C and D. A 10 μs production run was generated with the same settings as the equilibration, but with a time step of 25 fs and a metadynamics bias applied using PLUMED (32). Gaussian height was 0.8 kJ/mol, and Gaussian width was 0.1 nm. For efficiency, the Gaussians were deposited on a grid between 0 and 22 nm. Biasing potentials were deposited once every 4000 steps, corresponding to a deposition frequency of 0.01 ps^−1^. An additional wall bias, with a strength of 10,000 kJ/mol, was applied to ensure that the simulation did not visit unphysical conformations with NTD distances above 20 nm (Fig. S4), following the protocol for a study of conformational changes of the CorA magnesium transporter (36).

### Preparation of SANS data for use in BME (“*in silico* purification”)

SANS data were available from the small-angle scattering biological data bank (37) (SASBDB ID: SASDD26) and have previously been described and analyzed (12). The data contained a fraction of aggregates (12), which cannot be taken into account in the Bayesian/maximum entropy (BME) algorithm (38) (algorithm described below). Therefore, we used a novel post-processing protocol to be able to use the data.

• STEP 1. Choose a model for the aggregates.

The aggregates were described by a fractal structure factor with a dimensionality of two (structure factor *S*_2,D=2_ in Ref (39)).

• STEP 2. Fit the data with a fraction of aggregates.

Data were fitted with a combination of GluA2 tetramers and a fraction of aggregates of GluA2 tetramers, as in Ref (12). The fit had two *q*-dependent terms and a constant background (Fig. S5):

$$I_{\text{fit}}\;\left(q\right)\;=\;I_{\text{GluA}2\;\text{tetramer},\text{fit}}\;\left(q\right)\;+\;I_{\text{aggregate},\text{fit}}\;\left(q\right)\;+\;\text{background.}$$

where *I*(*q*) are the scattering intensities, which depend on the momentum transfer (*q)*. *q* is given in terms of the wavelength of the incoming light, λ, and the scattering angle, 2θ, *q* = 4π sin(θ)/λ. The model was implemented in WillItFit (40). The desensitized and NTD-splayed structure of GluA2 (EM-class 3, EMD-2688 (11)) was used to describe isolated GluA2 tetramers and its form factor was calculated with CaPP (https://github.com/Niels-Bohr-Institute-XNS-StructBiophys/CaPP).

• STEP 3. Subtract the aggregate term.

The fitted contribution from the aggregates was subtracted from the original experimental data to get a “filtered” dataset (Fig. S5):

$$I_{\text{filtered}}\;\left(q\right)\;=\;I_{\text{original}}\left(q\right)\;\;–\;\;I_{\text{aggregate},\text{fit}}\left(q\right)$$

The “filtered data,” *I*_filtered_(*q*), were used in the BME algorithm.

The method is subject to assumptions about the aggregated part of the sample (12, 39) and should therefore be used with care. However, if this filtering protocol had not been performed, the data would be systematically misleading and result in an ensemble with too many NTD-splayed structures, to compensate for the aggregates in the sample, which are not found in the simulated MD trajectory. We name the protocol “*in silico* purification” (and note that actual purification in the lab is always preferable). Albeit, non-ideal, the approach may be useful for other researchers, as many proteins are prone to aggregation (41), and much software for analysis of small-angle scattering data assumes homogeneous samples, *i.e.* samples without a fraction of aggregates (42).

### Calculating theoretical SANS scattering from the MD trajectory

The coarse-grained trajectory was converted to atomistic resolution using CG2AT2 (version 0.2) (43), with the *de novo* structures as output. 2000 frames were converted in total. Theoretical SANS curves were calculated from the atomistic structures using Pepsi-SANS (for Linux, version 3.0, https://team.inria.fr/nano-d/software/pepsi-sans/) (44). First, the curves were fitted against the filtered SANS data with all parameters free, and then they were all refitted with fixed global fitting parameters, following a previously established protocol (22). The hydration layer density was set to 5% higher than bulk water (45).

### Bayesian/maximum entropy (BME) reweighting

The initial weights from the metadynamics simulation were achieved using the PLUMED command REWEIGHT_BIAS. The metadynamics trajectory was reweighted using BME (version 2.0, in iterative mode, GitHub: sbottaro/BME2, downloaded 23.08.2022) (38). The filtered SANS data and the calculated SANS scattering from the simulated trajectory frames were used as input, as well as the initial weights for each frame, from the metadynamics coarse-grained MD simulations (Fig. S1). As an output from BME, a set of weights was given for varying values of the Lagrange multiplier, *θ* (38), which sets the minimum of the functional:

$$Q\;=\;\chi_{r}^{2}\;-\;\theta S_{\text{REL}}\text{.}$$

A value of *θ* = 1.0 was chosen, from a visual inspection of the goodness of fit ($\chi_{r}^{2}$) and the entropy term (*S*) as a function of *θ* (Fig. S6) (38). To assess what $\chi_{r}^{2}$ to aim for (which was determined to be 1.6 for the filtered SANS dataset), we used a Bayesian indirect Fourier Transformation (IFT) algorithm, BayesApp (version 1.0) (46, 47), with *D*_max_ = 200 and log(*α*) = 14 as initial guesses in the fit. 1000 extra error calculations were made to improve the error estimate and a constant background was fitted.

### Potential of mean force calculations

NTD domains from subunits B and D of GluA2 (Fig. 1) were isolated from the EM structure of full-length GluA2 in the compact desensitized state (PDB: 5VHZ (18)), using PyMOL (version 1.2, Schrödinger, LLC). The isolated domains consisted of residues Asn25 to Thr398 from subunits B and D. The structure was placed in a box with dimensions 25 × 15 × 15 nm^3^ (Fig. S3). For protonation assays, two structures were used, one with H229 protonated only at the N_E2_ site and one with H229 protonated also at the N_D1_ site. For GluA1, the NTDs of the crystal structure (PDB: 3SAJ (48)) were aligned to the NTD of desensitized GluA2 (PDB: 5VHZ (18)) and used as starting structure. GluA2-H229N and GluA1-N222H mutants were generated using the PyMOL mutagenesis tool. Simulations were run in GROMACS 2018 (31), with the CHARMM36 atomistic force field (49) with TIP3P water and ions added to neutralize the system. The system was first minimized, then equilibrated in the NVT ensemble and subsequently in the NPT ensemble; 100 ps in each ensemble and with a timestep of 2 fs. The LINCS algorithm was applied to constrain H-bonds. Verlet cutoff scheme was used with a cut-off distance of 1.2 nm. Electrostatics were calculated with Particle mesh Ewald, with a cut-off of 1.2 nm and PME order of 4. The temperature was kept constant at 300 K with a v-rescale thermostat applied to protein and solvent, respectively, with a time constant of 0.1 ps. The pressure was held at 1 bar with a Berendsen isotropic barostat with a 2 ps time constant and isothermal compressibility of 4.5⋅10^-5^ bar^-1^. Umbrella sampling was done using the pull code, starting from the equilibrated structures. First, a trajectory was made where the NTDs were pulled apart along the x-axis with a rate of 10 nm/ns and a force constant of 1000 kJ/mol/nm^2^. From this trajectory, frames were extracted with NTD distances spaced 0.1 nm apart, starting from the initial structure and ending with frames where the NTDs were 8 nm apart. Each frame was then simulated for 10 ns, with the NTD distance constrained, also using the pull code. These umbrella simulations were used to calculate the potential of mean force as a function of NTD distance, using the weighted histogram analysis method (50).

### Visualization of simulations

Simulation snapshots were retrieved in GROMACS and visualized with PyMOL. All plots were generated with Python 3, using NumPy (51) and Matplotlib (52).

### Probing protonation sites

We used the PDBePISA server (53) to map out interfacial amino acid residues between the interfaces, involved in salt bridges, which can be broken by protonation. PropKa (version 3.4.0) (54, 55) was used to estimate pKa values of the desensitized state (PDB: 5VHZ (18)) and the resting state (PDB: 4U2P (56)). We used the web interface (https://server.poissonboltzmann.org/), where PropKa3 is implemented as part of PDB2PQR in the APBS software suite (version 3.5.1) (57). The unaltered PDB files were given as input. The percent residues in the protonated state was calculated from the difference between pKa and pH using the Henderson-Hasselbalch equation.

### Electrophysiology constructs and transfection

HEK293T cells (ATCC, CRL-11268) were used to recombinantly express AMPAR subunits for outside-out patch recordings. For AMPARs, the flip variant was used for GluA1 and GluA2, where GluA2 was Q/R unedited (GluA2Q). Mutant receptors were generated using site-directed mutagenesis and verified by sequencing; residue numbering includes the signal peptide. Cells were plated at low density (1.6 × 10^4^ cells/ml) on poly-D-lysine-coated 35 mm dishes and transiently transfected 48 h post-plating using the calcium phosphate precipitation method. cDNAs were co-transfected with a plasmid encoding green fluorescent protein (GFP) to identify transfected cells. After 6 to 8 h, cells were washed twice with PBS and maintained in a fresh medium.

### Electrophysiology recordings and analysis

All recordings were performed 24 to 48 h post-transfection. The external solution contained (in mM): 150 NaCl, 5 HEPES, 0.1 CaCl_2_ and 0.1 MgCl_2_, and 2% phenol red at pH 7.4. For experiments at pH 5.5, 5M HCl was added to the external solution. The internal solution contained (in mM): 115 NaCl, 10 NaF, 5 HEPES, 5 Na_4_BAPTA, 0.5 CaCl_2_, 1 MgCl_2_ at pH 7.3 to 7.4. The osmotic pressure of all solutions was adjusted to 295 to 300 mOsm with sucrose. L-Glutamate was applied at 10 mM.

Recording pipettes were composed of borosilicate glass (3–6 MΩ, King Precision Glass, Inc) coated with dental wax. Agonist solution was rapidly applied using a piezo-stack driven perfusion system (Physik Instrumente), and solution exchange (<400 μs) was determined by measuring the liquid junction current at the end of each experiment. All recordings were performed using an Axopatch 200B amplifier (Molecular Devices, LLC). The holding potential during recordings was −60 mV. Current records were filtered at 5 kHz and sampled at 25 kHz. Series resistance (3–12 MΩ) was compensated for by 95%. All experiments were performed at room temperature. Data were acquired using pClamp9 software (Molecular Devices, LLC).

Electrophysiological recordings were analyzed using Clampfit 10.5 (Molecular Devices, LLC) and illustrated using Origin 2020 (OriginLab). Current decay rates were fit using first- or second-order exponential functions of the form $y=\sum_{i}A_{i}\cdot \exp\;\left(-x/\tau_{i}\right)$. For decay rates requiring second-order exponential fits, time constants are presented as weighted means. To measure recovery from desensitization, a two-pulse protocol (each 250 ms long) was used in which an agonist was applied at variable interpulse intervals, and the peak amplitude of the second (test) pulse was expressed as a fraction of the peak amplitude of the first (initial) pulse. The recovery time course for each patch was fit by a first-order exponential function, as described previously (58). Data are presented as mean ± SEM, where *n* values refer to the number of individual patches. Data were assessed for normality using a Shapiro-Wilks test and appropriate parametric or nonparametric tests were conducted accordingly, that is, paired/unpaired Student’s *t* test or Wilcoxon/Mann–Whitney *U* test. Significance was defined as *p* < 0.05. Statistical analysis was conducted using custom statistical software kindly provided by Dr Joe Rochford (McGill University).

## Data availability

Raw data is available upon request to the corresponding authors. Scripts for MD simulations and BME reweighting are available at https://github.com/andreashlarsen/Larsen2024-GluA2. Scripts for preparing the filtered SANS dataset, which was used in BME, are also available there.

## Supporting information

This article contains supporting information.

## Conflict of interest

The authors declare that they have no known competing financial interests or personal relationships that could have appeared to influence the work reported in this paper.
