# Supplementary material for: Recovery from desensitization in GluA2 AMPA receptors is affected by a single mutation in the N-terminal domain interface
Source: J Biol Chem. 2024 Feb 2;300(3):105717. doi: 10.1016/j.jbc.2024.105717 (PMC10909779; doi:10.1016/j.jbc.2024.105717)
Supplement: Supporting information [file mmc1.pdf]

Supporting Information for

Recovery from desensitization in GluA2 AMPA receptors is affected by a single mutation in the N-terminal domain interface

Andreas Haahr Larsen[1+*], Amanda M. Perozzo[2+], Philip C. Biggin[3], Derek Bowie[2] & Jette Sandholm Kastrup[4*]
[1] University of Copenhagen, Department of Neuroscience, Copenhagen, Denmark.
[2] McGill University, Department of Pharmacology and Therapeutics, Montreal, Canada.
[3] University of Oxford, Department of Biochemistry, Oxford, UK.
[4] University of Copenhagen, Department of Drug Design and Pharmacology, Copenhagen, Denmark.
[+] Contributed equally
[*]Corresponding authors: andreas.larsen@sund.ku.dk, jsk@sund.ku.dk

Content

- Video S1. Coarse-grained simulations with metadynamics. Screen grab and caption in this document, video in a separate file.
- Figure S1. Reweighting: weights and radius of gyration of frames
- Figure S2. NTD binding energy in GluA2 with fully protonated H229
  Figure S3. Simulation setup
- Figure S4. Coarse-grained metadynamics MD biases
- Figure S5. "*In silico* purification"
- Figure S6. Reweighting: balancing goodness of fit to SANS data and the entropy

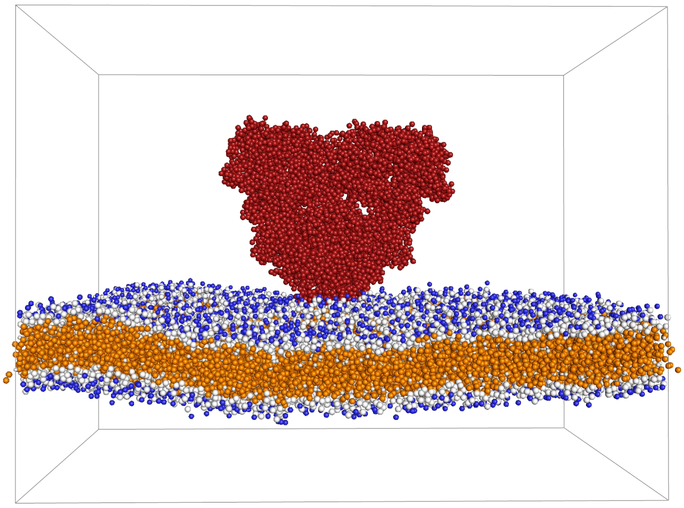

Video S1. Coarse-grained simulations with metadynamics. GluA2 homotetramer (red) in a POPC lipid bilayer (heads in blue/white and tails in orange), simulated for 10 μs. Water beads omitted for clarity. This is a screen grab from the video which is provided in a separate file.

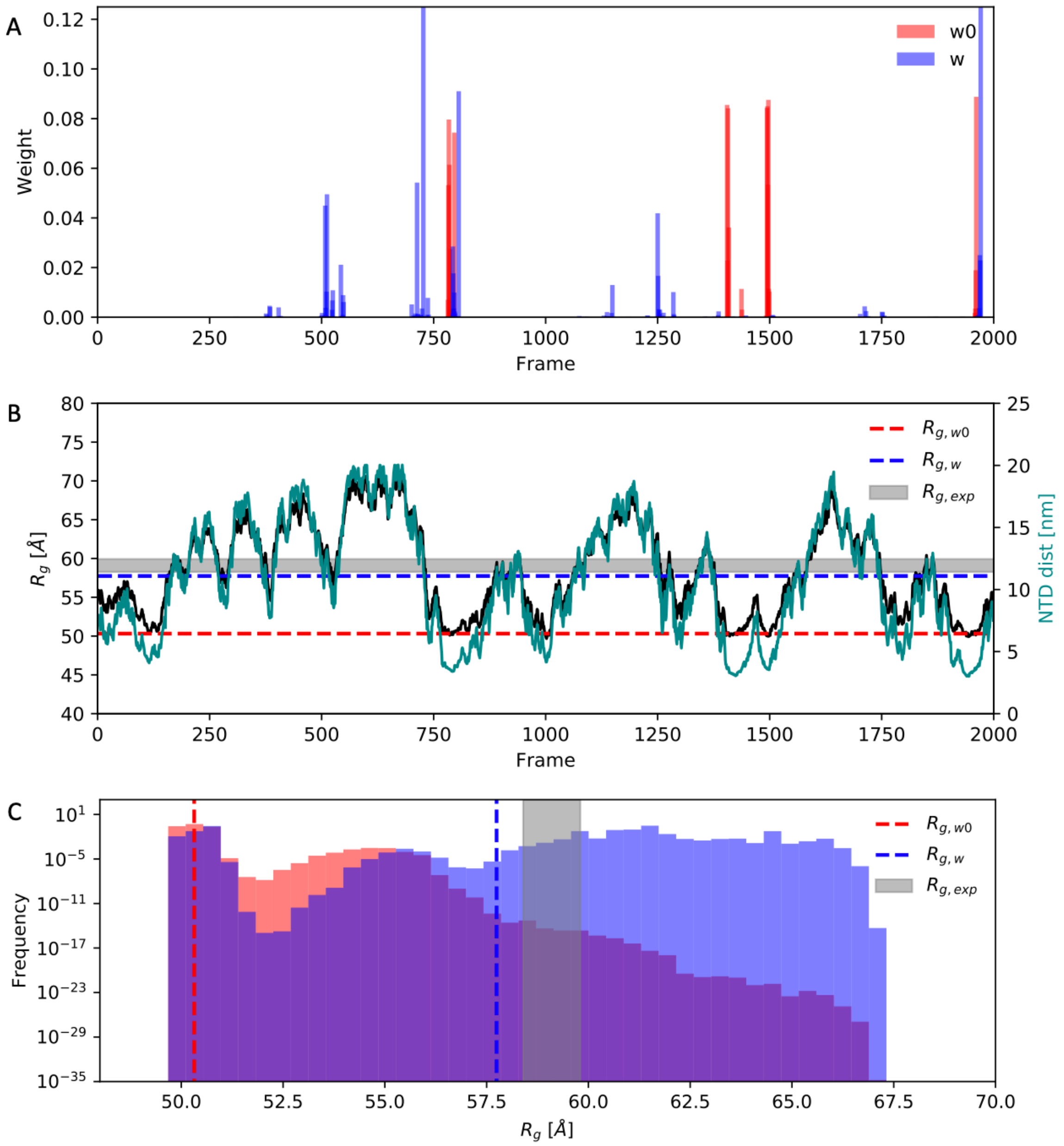

Figure S1. Reweighting: weights and radius of gyration of frames. (A) Weights before, w_0, and after, w, reweighting. Due to the metadynamics enhanced sampling technique, the initial weights are not uniformly distributed. (B) Corresponding values of R_g and NTD distances. (C) R_g distribution of ensemble before and after reweighting, plotted on log-y scale to highlight low-frequency states.

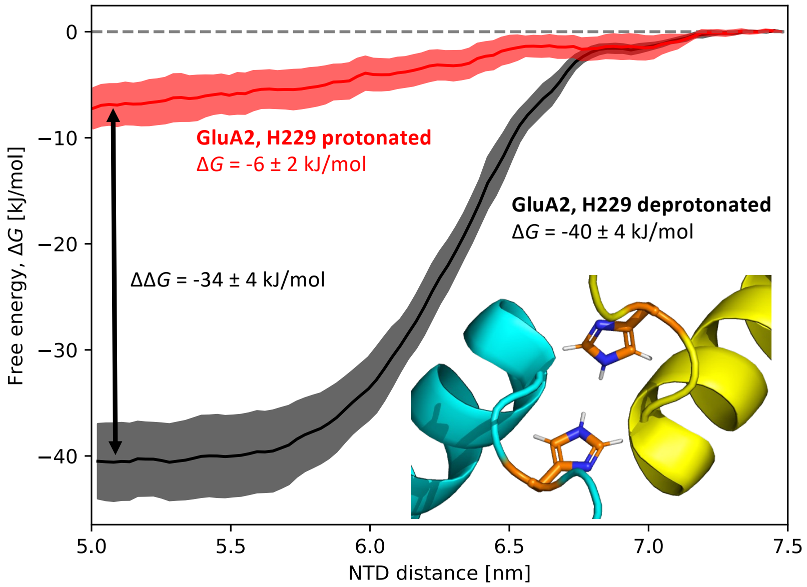

Figure S2. NTD binding energy in GluA2 with fully protonated H229. Free energy estimated by potential of mean force (mean and standard error of five repeats) for GluA2-NTD with H229 protonated (red) or deprotonated (black). Inset shows H229 in the NTD interface.

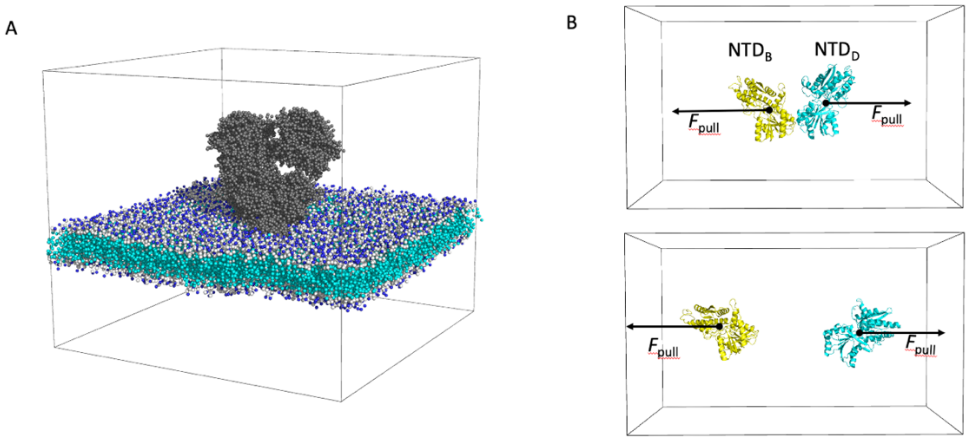

Figure S3. Simulation setup. (A) Coarse-grained simulation box after equilibration. (B) Atomistic simulation box for free energy calculations, with NTD$_B$ and NTD$_D$ together (top) and pulled a distance apart (bottom). Illustration of the effective pulling forces shown (arb size of the force vectors). Water and ions omitted for clarity in all panels.

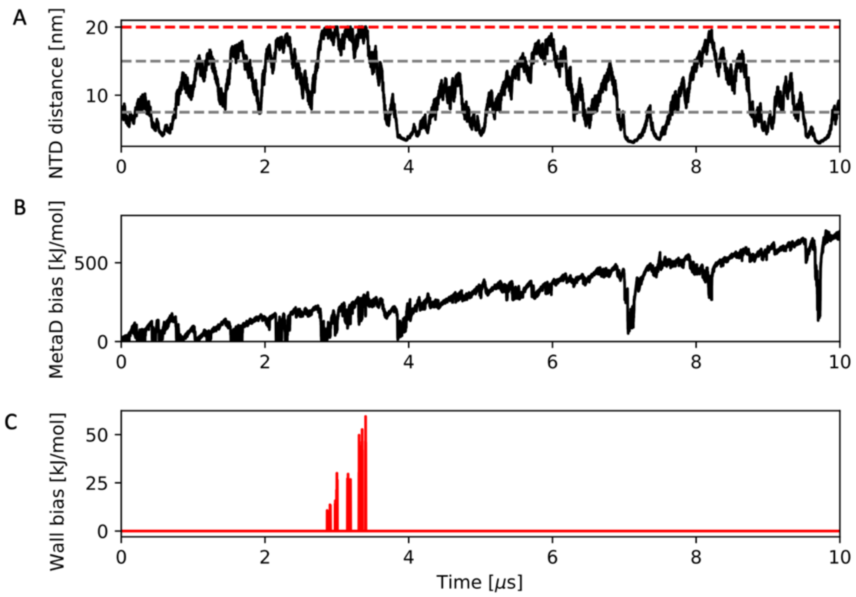

Figure S4. Coarse-grained metadynamics MD biases. (A) NTD distance. (B) Energy bias applied by the metadynamics algorithm. (C) Wall bias at 20 nm, preventing unphysical states.

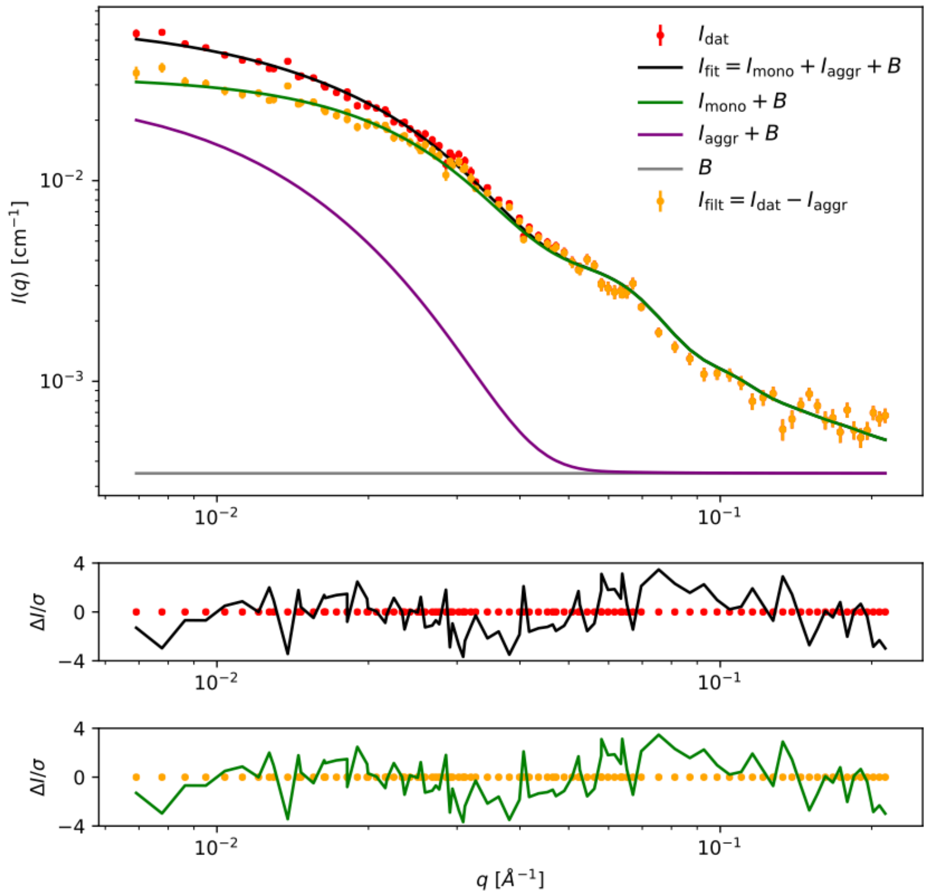

Figure S5. "*In silico* purification". The original SANS dataset (red) was fitted with a model of isolated GluA2 tetrameric molecules (green) and aggregates of GluA2 (violet) as well as a constant background (gray). The scattering contribution from aggregates were subtracted from the original data to obtain a "filtered" dataset. This filtered dataset can be fitted with scattering from GluA2 alone (without aggregates).

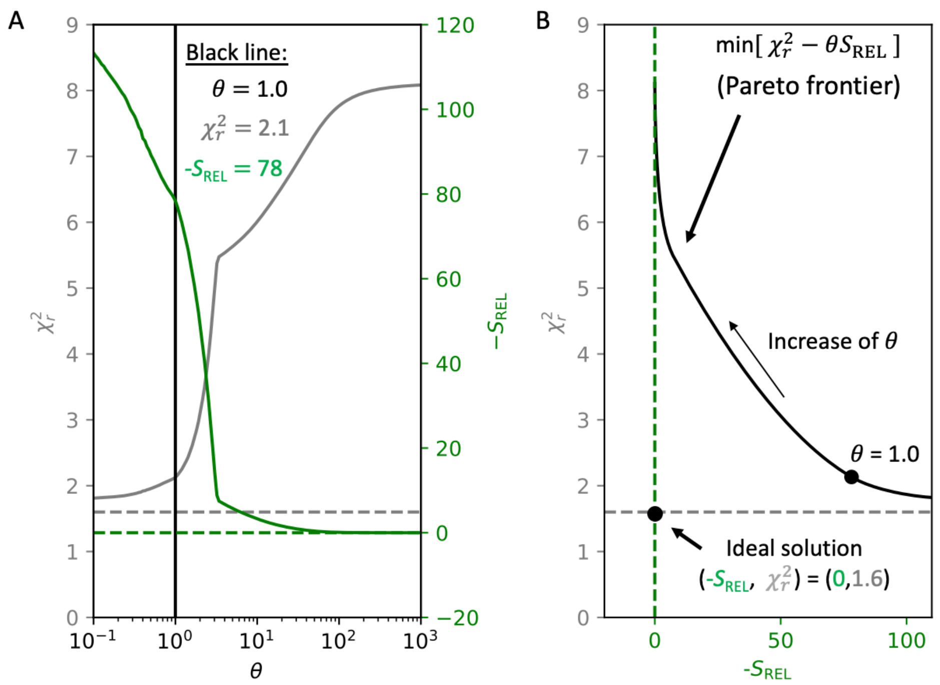

Figure S6. Reweighting: balancing goodness of fit to SANS data and the entropy. (A) In gray, the goodness of fit (χ_r^2) of the theoretical curve from the simulated data compared to experimental SANS data as θ is varied. Gray dashed line marks χ_r^2 = 1.6, the theoretical target value. In green, the entropy term, S_{REL}, penalizing deviation from the original simulated ensemble. The theoretical ideal value of S_{REL} is 0, and marked with a green dashed line. The best possible solution balance these contributions via θ, and values of χ_r^2 and S_{REL} at θ=1 are shown. (B) Pareto frontier plot, with the theoretical ideal solution marked as a dot. The best possible solution depends on the choice of θ, but it is always found on the Pareto frontier (black line), which is defined as the minimum of the functional χ_r^2 - θ S_{REL} for fixed values of θ.
